# The perspectives of NETosis on the progression of obesity and obesity-related diseases: mechanisms and applications

**DOI:** 10.3389/fcell.2023.1221361

**Published:** 2023-08-15

**Authors:** Jinyu Li, Lijia Yin, Siyi Chen, Zelin Li, Jiatong Ding, Jiaqiang Wu, Kangping Yang, Jixiong Xu

**Affiliations:** ^1^ Department of Endocrinology and Metabolism, First Affiliated Hospital of Nanchang University, Nanchang, China; ^2^ The First Clinical Medical College of Nanchang University, First Affiliated Hospital of Nanchang University, Nanchang, China; ^3^ The Second Clinical Medical College of Nanchang University, Second Affiliated Hospital of Nanchang University, Nanchang, China; ^4^ Jiangxi Clinical Research Center for Endocrine and Metabolic Disease, Nanchang, Jiangxi, China; ^5^ Jiangxi Branch of National Clinical Research Center for Metabolic Disease, Nanchang, Jiangxi, China

**Keywords:** neutrophil extracellular trap networks, NETosis, obesity, nonalcoholic steatohepatitis, diabetes, atherosclerosis

## Abstract

Obesity is a disease commonly associated with urbanization and can also be characterized as a systemic, chronic metabolic condition resulting from an imbalance between energy intake and expenditure. The World Health Organization (WHO) has identified obesity as the most serious chronic disease that is increasingly prevalent in the world population. If left untreated, it can lead to dangerous health issues such as hypertension, hyperglycemia, hyperlipidemia, hyperuricemia, nonalcoholic steatohepatitis, atherosclerosis, and vulnerability to cardiovascular and cerebrovascular events. The specific mechanisms by which obesity affects the development of these diseases can be refined to the effect on immune cells. Existing studies have shown that the development of obesity and its associated diseases is closely related to the balance or lack thereof in the number and function of various immune cells, of which neutrophils are the most abundant immune cells in humans, infiltrating and accumulating in the adipose tissues of obese individuals, whereas NETosis, as a newly discovered type of neutrophil-related cell death, its role in the development of obesity and related diseases is increasingly emphasized. The article reviews the significant role that NETosis plays in the development of obesity and related diseases, such as diabetes and its complications. It discusses the epidemiology and negative impacts of obesity, explains the mechanisms of NETosis, and examines its potential as a targeted drug to treat obesity and associated ailments.

## 1 Introduction

Currently, obesity has replaced malnutrition and diseases caused by infection as diseases with the highest mortality from its complications. Recent statistics indicate that the number of overweight or obese individuals globally continues to escalate, with over 200 million affected individuals, which accounts for approximately 30% of the world’s population. The Global Burden of Disease Panel reported that the prevalence of obesity had doubled in more than 70 countries since 1980 and continued to increase in most other countries in 2017 (Collaborators et al., 2017).

Studies have now been conducted that link obesity to a variety of diseases and related complications, such as nonalcoholic fatty liver disease, type 2 diabetes, and atherosclerosis. Obesity not only has a serious impact on people’s quality of life but can also have a negative impact on mental health. With obesity escalating at an alarming rate, it is imperative to conduct more research into the mechanisms that drive obesity to facilitate the development of preventative and remedial strategies for obesity and related health conditions.

Multiple studies have found that obesity-related diseases lead to an abnormal immune system and cell population (Fang et al., 2020a; Hildreth et al., 2021), such as CD4, CD8 (Yang et al., 2010), Th17, Th22 (Fabbrini et al., 2013), mononucleosis (Ilavská et al., 2012), macrophages (Wu et al., 2007), eosinophils (Moussa et al., 2019), neutrophils, and pathological enrichment of immune cell subsets. Among them, neutrophils play an important role in human immunity, which is an important role for the body to clear foreign threats and autoimmunity. The death modes of central granulocytes include pathogen-induced cell death, necrosis, pyrodeath, autophagy, aging and NETosis. NETosis is a specific type of neutrophil death mechanism that is different from apoptosis and pyrodeath. Its primary role is to enhance organismal immunity by lysing the nuclear membrane, decondensing DNA, rupturing the plasma membrane, and releasing reticular structures to capture and eliminate pathogens. Multiple studies have demonstrated the involvement of NETosis in the mechanisms and progression of various obesity-related diseases.

This review aims to provide an overview of the mechanisms of NETosis and its positive effects on obesity and related diseases. It will also list drugs commonly used in these diseases to guide future research on the mechanisms, as well as on the diagnosis and treatment of obesity and its complications.

## 2 Mechanisms of NETosis

### 2.1 Pathophysiological mechanism of NETosis

**FIGURE 1:**
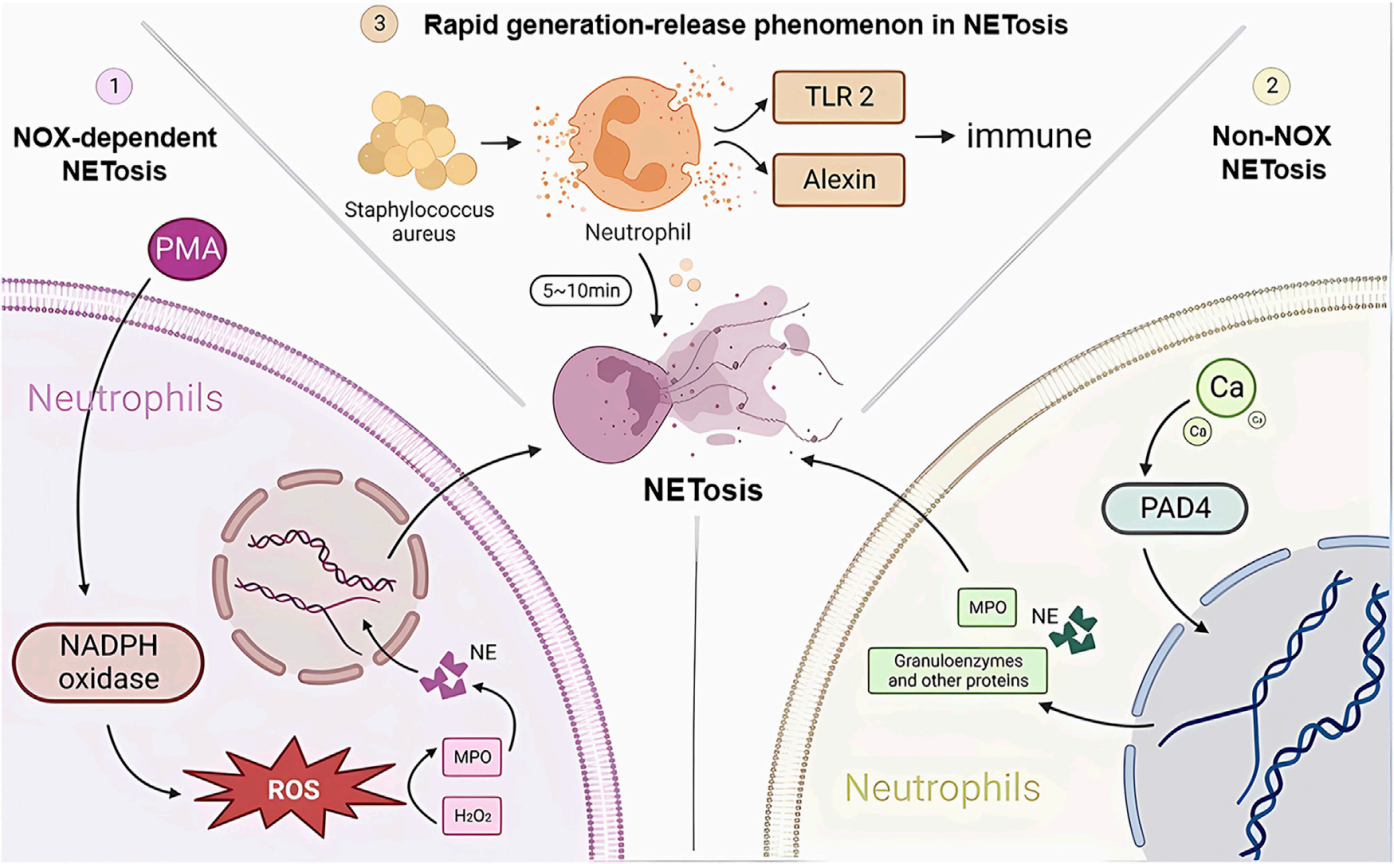
NETosis developmental mechanisms. Three different pathways of NETosis development are plotted in the figure. NADPH, nicotinamide adenine dinucleotide phosphate; NE, neutrophil elastase; MPO, myeloperoxidase; H2O2, hydrogen peroxide; TLR2, Toll-like receptor 2; PAD4, peptidylarginine deaminase 4.

Neutrophils are the first immune cells recruited to the relevant site when the body is exposed to infection or injury and are the first line of defense against foreign microorganisms, playing an important role in internal immunity. Neutrophil extracellular trap networks (NETs) are a recently discovered anti-inflammatory mechanism. Unlike apoptosis and necrosis, NETosis is a special form of programmed cell death that is released when neutrophils are activated (Takei et al., 1996; Brinkmann et al., 2004), a process of NET release known as NETosis. In this process, the nucleus of the neutrophil loses its identity, its chromatin is stripped, and the nuclear membrane and particles breakdown and mix together. NET cells are subsequently released extracellularly by neutrophils and consist mainly of a double-stranded DNA complex, a reticulum complex consisting mainly of double-stranded DNA, along with a large number of granular proteins and various anti-inflammatory proteins, such as antimicrobial peptides, proteases, and guanosine histones (Fousert et al., 2020). The amount of each protein component depends on the proportion of NETs released by different neutrophil subpopulations (Pinegin et al., 2015; Hamam and Palaniyar, 2019). In contrast to other cell death processes, such as apoptosis, necrosis, or apoptosis, chromatin deperfusion is the main defining feature of NETosis. Chromatin decompression is thought to be mediated by histone posttranslational modifications, the most typical of which are PAD4-mediated citrullination and serine proteinase-mediated cleavage (Wong and Wagner, 2018). The study found that there are three main types of activation mechanisms during the anti-inflammatory immune response to NETosis (Figure 1). One is NOX-dependent NETosis, a collective term for the catalytic subunit of NADPH oxidase gp91phox/NOX2 and its homologs, where the key step in NETosis is the production of ROS by NADPH oxidase (Fousert et al., 2020). The most potent inducer is fosfomycin myristate (PMA) (de Bont et al., 2018). In addition to killing microorganisms, ROS also play an important role in the subsequent production of NETs, creating the conditions for the subsequent activation of myeloperoxidase (MPO) by H2O2 as a substrate, which in turn induces the release of neutrophil elastase (NE). Optimal conditions and environmental pH. After 60 min of stimulation, HOCI breaks down the cytoplasm and releases activated NE into the cytoplasm. One hundred 20 minutes later, NE degrades F-actin and enters the nucleus, breaking down histone H1 and finally reaching the core histones. Later, MPO, together with NE, promotes histone decondensation, the unfolding of additional chromatin and further disruption of the nuclear membrane. The nuclear membrane ruptures, gradually disintegrating into vesicles and releasing them into the cytoplasm, allowing the cytoplasm to mix with the nucleoplasm and decondensed chromatin to bind to cytoplasmic antimicrobial proteins, granulins and other proteins. Finally, with the rupture of the plasma membrane, NETs are released, followed by neutrophil death (Amulic et al., 2017; Bravo-Barrera et al., 2017; Manfredi et al., 2018; Volkman et al., 2019).

Another type of NETosis does not depend on NADPH, and the occurrence of non-NOX-dependent NETosis depends on calcium inward flow, where the calcium carrier ionomycin, A23187, and pH elevation can induce non-NOX-dependent NETosis (Ravindran et al., 2019). As the concentration of calcium ions in the cytoplasm rises, peptidylarginine deaminase 4 (PAD4) activates, guanylating histones (mainly H3) and reducing the positive charge carrying capacity of histones, resulting in a weakened ability to bind proteins to DNA, thus causing chromatin decondensation. Chromatin decondenses into the cytoplasm and binds granzymes such as MPO, NE and other proteins, which are eventually released into the extracellular space to form NETs (Eghbalzadeh et al., 2019; Holmes et al., 2019). It has been shown that cells deprived of PAD4 will not be able to successfully release NETs (Sorensen and Borregaard, 2016). It has also been shown that in resting neutrophils, PAD4 is associated with the cytoplasmic subunit of NADPH oxidase and that this association affects ROS production. Thus, PAD4 may play a different and important role in NOX-dependent *versus* non-NOX-dependent NETosis (Zhou et al., 2018).

**FIGURE 2:**
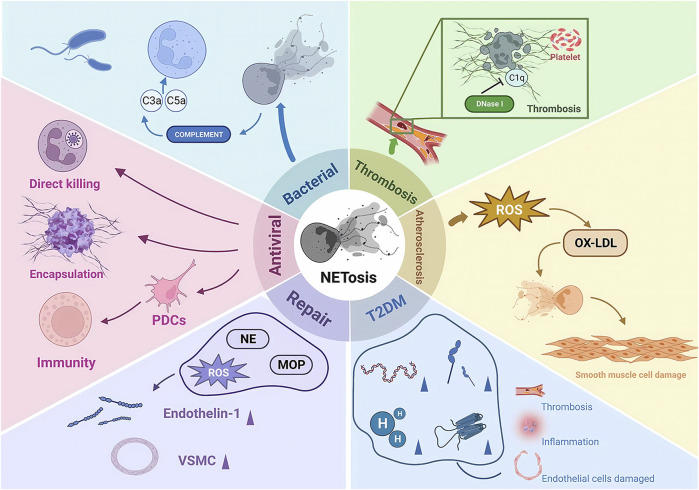
The pathophysiological role of NETosis. NE, neutrophil elastase; MOP, myeloperoxidase; ROS, reactive oxygen species; PDCs, plasmacytoid dendritic cells; ET1, endothelin 1; VSMCs, vascular smooth muscle cells; IL-6, interleukin 6; OX-LDL, oxidized low-density lipoprotein; DNase 1, deoxyribonuclease I.

There is another rapid generation-release phenomenon in NETosis. In nitrogen-dependent and nonnitrogen-dependent cases, NET release takes 3–4 h (Ahmad et al., 2021) and eventually leads to death. In contrast, recent studies have shown that neutrophil skin infections such as *Staphylococcus aureus* produce within 10 min of stimulation, and even when NETosis occurs, some neutrophils do not die but remain active, showing an immune response through crawling Toll-like receptor 2 (TLR2) and the protein of the compliment, a granular release network that extends the unstable pseudopod and extends it through the skin. Transiently nucleated gran neutrophils are left behind and undergo hyperpolarization, which is the phenomenon of rapid opening of ion channels on the neutrophil cell membrane and a rapid increase in cell membrane potential, which changes the content of avidin on the cell membrane and promotes the secretion of microbial killer molecules, forming NETs (Hilscher and Shah, 2020; Nija et al., 2020; Alasmari, 2020). The rapid formation of NETs may also be an important mechanism for fighting infection, which may primarily act in the early stages of infection. In the human immune system, NETosis is a double-edged sword. On the one hand, NETosis has been described as a neutrophil defense mechanism of the organism that traps and destroys a wide range of pathogens involved in anti-inflammatory, antibacterial and antiviral immune responses, including Gram-positive bacteria (von Kockritz-Blickwede and Winstel, 2022), Gram-negative bacteria and their virulence factors (Sung et al., 2022), fungi (Huang et al., 2022), protozoa (Conejeros et al., 2022) and viruses (Kapoor and Shukla, 2023). NETosis is also involved in the normal regeneration and repair of blood vessels. On the other hand, there is increasing evidence from recent studies that NETosis is also involved in the pathophysiology of many diseases, such as autoimmune diseases (Alaygut et al., 2023), diabetes (Farhan et al., 2023), cardiovascular diseases (Fadini et al., 2016b), cancer (Tomas-Perez et al., 2023) and Alzheimer’s disease (Smyth et al., 2022). Overactivation and inappropriate recruitment of NETosis may lead to severe organismal damage and disease deterioration (Figure 2).

Recent studies have also shown that NETosis is biased and that there is a specific subpopulation bias in the formation of NETosis by neutrophils (Denny et al., 2010; Ng et al., 2019). In some autoimmune diseases, such as systemic lupus erythematosus (SLE), the phenomenon of NETs resulting from an increase in this class of neutrophils may be a potential mechanism for the development of symptoms and complications of autoimmune diseases (Kaplan, 2011; Villanueva et al., 2011; Bengtsson et al., 2014; Wirestam et al., 2019). During the clearance of infection and immune defense by NETs, neutrophils also inevitably release their own antigens extracellularly, bringing a range of products into frequent contact with the body’s immune system and greatly increasing the chances of possible autoimmunity, making them an important target for immune responses. In addition, the large number of active mediators released by NETs, if combined with proinflammatory factors, will accelerate the inflammatory process and intensify the immune response (Lee et al., 2017b; Jorch and Kubes, 2017; He et al., 2018; Fousert et al., 2020).

It is worth noting that NETosis may possess both anti-inflammatory and proinflammatory effects, which are mainly related to the timing and concentration of NETs (Schauer et al., 2014). The mediators released by NETs in the early stages of inflammation contribute to the recruitment and attachment of platelets, macrophages and neutrophils, promoting an inflammatory immune response. In contrast, the dense reticulum structure in later stages may serve to confine areas of inflammation, and serine proteases attached to the reticulum are able to block neutrophil chemotaxis and contribute to the regression of inflammation by degrading inflammatory factors such as IL-1β and chemokines (Joosten et al., 2010; Mitroulis et al., 2011). Two conditions are necessary for the onset of acute gout: monosodium urate (MSU) deposition and the body’s inflammatory response to crystals. Both of these utilities play a significant role in the onset and spontaneous remission of acute gout (Steiger and Harper, 2014; Dalbeth et al., 2021).

In the context of the proinflammatory and prothrombotic phenomenon of excessive NETosis, several studies have shown the involvement of NETosis in the development and progression of many disease complications (Fadini et al., 2016b; Smyth et al., 2022; Alaygut et al., 2023; Farhan et al., 2023; Tomas-Perez et al., 2023). It has been suggested that the hyperglycemic environment of diabetic patients may induce the formation of NETs and NETosis (Menegazzo et al., 2015). In addition, patients with type 2 diabetes have increased circulating NETosis products such as histones, DNA, elastase and circulating mono- and oligonucleosomes and produce more IL-6 (Avogaro et al., 2003). This set of changes may lead to thrombosis, inflammation and endothelial dysfunction, which can affect the development of diabetic complications such as diabetic macular degeneration, diabetic nephropathy, cardiovascular disease and diabetic foot. The mechanism by which the high-glucose environment promotes the development of NETosis is not yet clear but may be related to the glucose-induced increase in NADPH oxidase, which requires further study (Almyroudis et al., 2013; Keshari et al., 2013).

### 2.2 Detection method

Currently, fluorescence microscopy is the most widely accepted method for visualizing and quantifying NETs. However, this approach has its drawbacks; it is subjective, time-consuming, and yields a small sample size of analyzed polymorphonuclear cells (PMN) per sample. There is now a growing interest in using flow cytometry techniques to identify NETs. However, flow cytometry analysis of NETs requires special attention to sample preparation to obtain reproducible data. The assay uses a combination of fluorescently labeled antibodies against cellular markers on PMNs and nucleic acid stains to measure NETosis in whole blood (WB) and purified PMNs using the plasma membrane impermeable DNA binding dye SYTOX Orange (SO), which is positive for SO and DAPI in the cell-attached DNA of NETting PMNs. The combination of optimally diluted antibody and nucleic acid dye requires no washing and produces low background fluorescence. Because WB and purified PMNs correlate significantly with NETosis, the assay can be validated by comparison with time-lapse live cell fluorescence microscopy. The assay can be well applied to diseases associated with NETosis, such as systemic lupus erythematosus (SLE). The assay is observer-independent and allows rapid assessment of large numbers of PMNs per sample, does not require sophisticated microscopic equipment such as imaging flow cytometry, and can be used as a starting point for analyzing the formation of extracellular traps (traps) in immune cells other than PMNs (Zharkova et al., 2019). Another novel imaging flow cytometry (IFC) allows specific detection and quantification of histone 4 (H4cit3) guanylation as a marker of NETs in whole neutrophils before the release of DNA and cytoplasmic protein chains into the extracellular space. Other analytical parameters looking at nuclear and cellular morphological changes (nuclear decondensation and hypercontraction, lobulated nuclei and cell membrane damage) can provide additional information about the behavior of the analyzed neutrophil population (Barbu et al., 2021). Another representative innovative and efficient analytical method is the imaging and computational algorithm of the High Context Screening (HCS) cytomics platform, which employs membrane permeable and impermeable DNA dyes to identify NET-forming cells *in situ*. Automated algorithm-driven single-cell analysis of nuclear morphology changes, nuclear area increases and intensity changes provides accurate detection of NET-forming cells and eliminates user bias against other cell death patterns. It is further combined with *in situ* staining for membrane-linked protein V to detect specific death pathways, such as apoptosis, and thus distinguishes between NET, apoptosis and necrosis. The method does not utilize fixation and permeabilization steps that interfere with NETs, thus allowing monitoring of NETs over time, and this imaging-based method for specific high-throughput NET analysis may provide a good platform for discovering potential inhibitors of NET formation and/or drugs that regulate neutrophil death (e.g., NETosis-apoptosis switch) as an alternative strategy for enhancing inflammatory resolution (Singhal et al., 2022). In addition, convolutional neural networks (CNNs) can also be used to accurately quantify neutrophil NETosis. CNNs have achieved >94% performance accuracy in distinguishing NETosis from non-NETosis cells and have greatly facilitated dose‒response analysis of NETosis responses in patients’ neutrophils and screening. Using only features learned from nuclear morphology, the CNN can distinguish between NETosis and necrosis and different NETosis signaling pathways, making it an accurate tool for NETosis detection (Elsherif et al., 2019). Other detection methods include ELISA, immunoblotting, and image flow-based methods, but all of them exhibit the disadvantages of subjectivity, nonspecificity, error proneness and low throughput.

## 3 NETosis in obesity and obesity-related diseases

### 3.1 Obesity

Before fully describing the relationship between obesity and NETosis, it is important to note that obesity is a heterogeneous disease, with variations in definitions of obesity and assessment criteria across populations and assessment methods. This heterogeneity can be explored through body mass index (BMI), waist circumference, and other relevant measures, and the potential impact of obesity on different disease mechanisms and the development of various conditions (e.g., nonalcoholic fatty liver disease, type 2 diabetes mellitus, and atherosclerosis) can be discussed. Body mass index (BMI), calculated by dividing body weight (kilograms) by the square of height (meters), is a commonly used indicator to define obesity. BMI is the most basic way of defining obesity; however, there is a U- or J-shaped relationship between BMI-defined obesity and mortality, which has led to the creation of other measures (Flegal et al., 2013). Waist circumference (WC) is another indicator used to assess central obesity, which is strongly associated with metabolic complications, which may be related to the fact that WC is more able to characterize the fat levels of visceral tissues (Armstrong et al., 2022).

In addition to BMI and WC, a number of other measures, such as waist-to-hip ratio (WHtR) and fat mass index (FMI), have also been used to define obesity. The waist-to-height ratio (WHtR) was shown to be superior to WC and BMI in detecting cardiometabolic risk factors in both sexes in multiple cohorts of large samples (Ashwell et al., 2012). A research trial showed that fat mass index (FMI) and WC were more useful in identifying the risk of obesity-associated neuropathy compared to BMI in patients with T2DM (Gao et al., 2021). The diversity of obesity definitions and measurements can greatly influence our understanding of disease mechanisms and associated disease progression. For example, differences in obesity criteria may influence the prevalence and severity of NAFLD, type 2 diabetes, and atherosclerosis. By exploring these associations, we can gain insight into potential interventions and personalized treatment strategies for obesity-related diseases.

The different measures also imply that the development of obesity is influenced by a variety of factors. The prevailing view is that chronic energy imbalance is the main factor in the development of obesity (Rohm et al., 2022). Obesity occurs when caloric expenditure in the body decreases and most of the energy is stored in fat cells as triglycerides. Triglycerides continue to accumulate in adipocytes, which limits blood flow in adipose tissue, and the oxygen supply is reduced as a result (Maurizi et al., 2018). As obesity progresses, intestinal mucosal permeability increases, and under the influence of a variety of factors, including intestinal microbes and their metabolites, obese patients develop mild chronic inflammation throughout the body.

Thus, the molecular and cellular mechanisms by which obesity leads to inflammation and insulin resistance involve several complex processes: 1) Release of inflammatory factors by adipocytes: adipocytes release a variety of inflammatory factors, such as tumor necrosis factor-alpha (TNF-α), interleukin-6 (IL-6), and C-reactive protein (CRP). These inflammatory factors can stimulate the inflammatory response and interfere with insulin signaling, leading to insulin resistance (Ahmed et al., 2021). 2) Oxidative stress in adipose tissue: oxidative stress can lead to the production of excessive reactive oxides in the cells, which can damage cellular structure and function and in turn trigger the inflammatory response and insulin resistance (Ouchi et al., 2011). 3) Lipid accumulation in adipose tissue: lipid accumulation can lead to the abnormal function of adipocytes, which release more inflammatory factors and interfere with insulin signaling, leading to insulin resistance. 4) Inflammatory cell infiltration in adipose tissue: the number of inflammatory cells such as neutrophils, macrophages and T-lymphocytes increases. These inflammatory cells release inflammatory factors, creating an inflammatory environment that further exacerbates insulin resistance (De Lorenzo et al., 2016). It is currently believed that in the early stages of obesity, adipose tissue triggers an inflammatory response, leading to the activation of inflammatory cells (e.g., macrophages and neutrophils) and the release of NETs, which induces NETosis. The formation of NETs may further exacerbate the inflammatory response and insulin resistance, creating a vicious cycle. Obesity is associated with oxidative stress and lipid accumulation, which may also be involved in the induction of NETosis.

**FIGURE 3:**
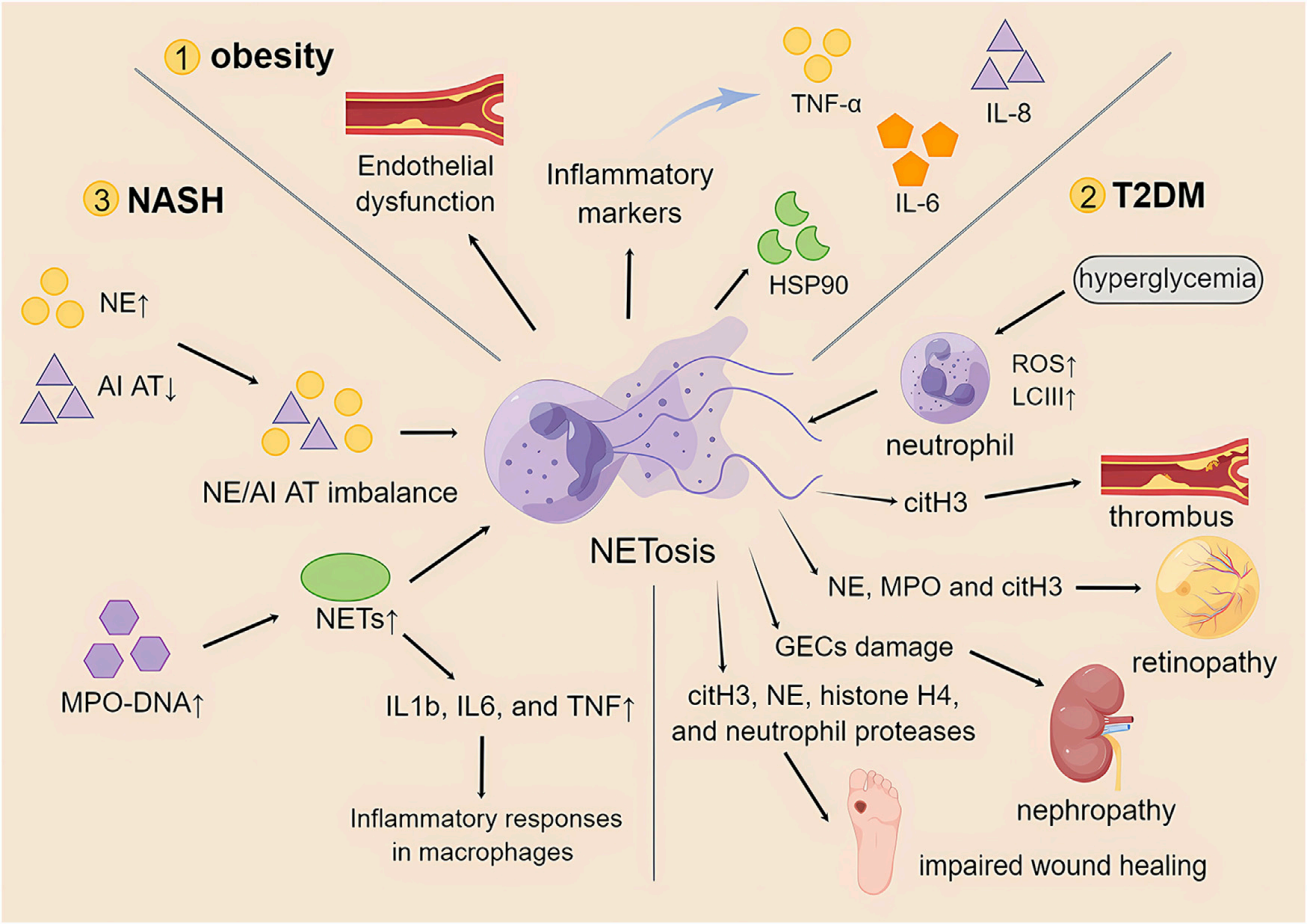
The role of NETosis in obesity and its associated metabolic diseases. TNF-α, tumor necrosis factor-α; IL-6, interleukin-6; IL-8, interleukin-8; HSP90, heat shock protein 90; T2DM, type 2 diabetes mellitus; ROS, reactive oxygen species; LCIII, microtubule-associated protein light chain III; citH3, citrullinated histone 3; NE, neutrophil elastase; MPO, myeloperoxidase; GECs, glomerular endothelial cells; NASH, nonalcoholic steatohepatitis; AI AT, α1-antitrypsin; NETs, neutrophil extracellular traps; IL1b, interleukin 1b.

Studies have shown that obesity increases the number of low-density neutrophils with inflammatory genetic features (Sanchez-Pino et al., 2022). This low-density neutrophil population showed proinflammatory, degranulation, and immunosuppressive functions. Furthermore, the study also showed that bariatric surgery, as an effective treatment for severe obesity, can reduce circulating LDNs and improve immunity and metabolism in obese patients. This also provides another idea for research: to improve multiple adverse effects in obese patients by modulating NETosis (Metzemaekers et al., 2020). Neutrophils are immune cells that infiltrate adipose tissue. The low-grade chronic inflammation produced by obesity plays an important role in the activation of neutrophils, and its mechanism and role in the formation of NETosis remain to be explored in greater depth. However, the findings regarding the mechanisms of NETosis in obesity are unclear or even contradictory (Figure 3).

Several studies point to an association of obesity-induced inflammation with high levels of NETosis. In a study in a high-fat diet (HFD) mouse model of obesity, endothelial dysfunction and high expression of NETosis in the mesenteric artery wall were reported (Wang et al., 2018a). When NETosis was suppressed, endothelial function was subsequently restored. Therefore, the occurrence of NETosis in obesity may be responsible for endothelial dysfunction. Recent database analysis studies have also revealed a strong relationship between inflammatory markers such as TNF-α, IL-6, IL-8, and heat shock protein 90 (HSP90) and NETosis formation in obese patients (Valeria Oliveira de Sousa et al., 2021; Freitas et al., 2022). It has also been reported that obese patients can reduce NETosis levels and thus improve the occurrence of adverse events through exercise (Valeria Oliveira de Sousa et al., 2021).

However, in other studies, low levels of NETosis were instead demonstrated in obese patients. A study that examined purified neutrophils from obese mice found that although neutrophils exhibited elevated ROS expression, suggesting neutrophil activation, they had lower NETosis than nonobese mice (Roberts et al., 2018). Similarly, when the hepatic vascular system of HFD mice was observed using *in vivo* microscopy, it was found that NETosis levels in obese mice remained lower than normal (Cichon et al., 2021).

Another class of studies notes that the direct causal relationship between NETosis and obesity is unclear. There is even an early study showing that inhibition of NETosis formation may not be clinically relevant to obesity-mediated adverse events such as adipose tissue inflammation and insulin resistance. A recent study has shown that severe obesity is associated with increased production of NETs (Rosales et al., 2017) and that NETosis in turn alters the systemic inflammatory status of patients (Rhee et al., 2018). Although bariatric surgery removed some of the adipose tissue, it failed to correct NETosis. Moreover, patients with the highest risk of cardiovascular events are suspected to be associated with the persistence of NETosis (D'Abbondanza et al., 2019).

The differences between the above studies may be related to the fact that different methods of assessing NETosis were used. Most of them used the following methods: a) detection of some NET components in plasma or serum; b) direct *in vitro* assessment of NETosis formation with purified neutrophils; and c) live microscopy to detect NETosis (none of them even confirmed the occurrence of NETosis). Elevated circulating DNA or neutrophil granulocyte protein does not equate to NETosis, and *in vitro* assays using purified neutrophils are more reliable. The results and conclusions are not only influenced by the limitations of the methods used but likewise depend on the differences in the study population. Therefore, future studies will require more rigorous assays and modeling criteria.

### 3.2 Type 2 diabetes

Type 2 diabetes mellitus (T2DM) is a chronic multifactorial disease closely associated with overweight and obesity. Its characteristic is relative insulin deficiency caused by pancreatic beta-cell dysfunction and target organ insulin resistance. In the presence of *ß*-cell dysfunction, insulin secretion is unable to maintain glucose homeostasis, resulting in abnormally high glucose levels in the blood (Kahn et al., 1993; Stumvoll et al., 2005). On the other hand, insulin resistance increases glucose production in the liver and decreases glucose uptake in skeletal muscle, liver, and adipose tissue (Petersen and Shulman, 2002; Leclercq et al., 2007; Czech, 2020). When these two pathological states coexist, they interact and may synergistically exacerbate T2DM (Cerf, 2013). Neutrophil elastase (NE) is a key effector involved in the inflammatory response to obesity, and its additivity in hepatocytes or adipocytes leads to cellular insulin resistance (Talukdar et al., 2012). In addition, the absence of NE leads to less tissue inflammation accompanied by improved glucose tolerance and increased insulin sensitivity. These results indicate that neutrophils may be directly involved in inflammation-induced metabolic diseases such as T2DM (Talukdar et al., 2012). Neutrophils may function as amplifiers of inflammatory signals and chemotactic agents for other inflammatory subpopulations, playing a crucial role in the regulation of insulin resistance in insulin-sensitive tissues.

Extensive evidence indicates the production of NETs and the circulation of NET markers, including nucleosomal DNA, HNE-DNA complexes, MPO-DNA complexes, and cell-free DNA (cfDNA). HNE and the expression of PAD4 on neutrophils are increased in patients with T2DM (Menegazzo et al., 2015; Wong et al., 2015; Carestia et al., 2016; Miyoshi et al., 2016; Wang et al., 2018b). A study found that the percentage of glucose-controlled markers HbA1c was positively correlated with circulating NET markers, including mononucleosomes and oligonucleotides. Hyperglycemia increased the release of circulating NET markers and NETosis. This finding provides evidence for a link between diabetes and NETs (Menegazzo et al., 2015). In addition, it has been demonstrated that hyperglycemia leads to increased levels of ROS in neutrophils of diabetic patients, which leads to an increase in LCIII, a marker of autophagy, and downstream NETosis (Farhan et al., 2023).

However, the role of NETosis in the pathogenesis of T2DM remains debated. A recent study provided evidence that increased formation of NETs (especially PAD4) is strongly associated with leaky gut in T1DM but unrelated to T2DM (You et al., 2019). A report showed impaired NET production under hyperglycemic conditions *in vitro* (Joshi et al., 2013). To explore the link between glycemic control and NETosis in T2DM patients, researchers found that increased NETosis in T2DM patients appeared to be due to proinflammatory cytokines rather than the result of impaired glycemic control when assessing whether NET levels changed after treatment with diabetic medications (Carestia et al., 2016).

Although the role of NETosis in the pathogenesis of T2DM remains controversial, there is growing evidence that NETs play an important role in diabetes-related complications. The presence of NETs in atherosclerotic plaques in mice and humans was demonstrated a decade ago (Megens et al., 2012), and a subsequent study showed that the NET component is significantly higher in patients with severe coronary artery disease (CAD) than in healthy controls (Borissoff et al., 2013) and that NETs may also impair plaque dissolution (Josefs et al., 2020), but the clinical use of NET markers in diabetic patients at risk of CAD has not been proven. By immunohistochemical staining analysis, citrullinated histone 3 (citH3), a marker of NETs, was detected in thrombi of almost all ischemic stroke patients (Laridan et al., 2017), and increased NETs in thrombus and peri-infarct brain tissue were associated with hyperglycemia (Deng et al., 2020).

Circulating DNA-histone complexes and polymorphonuclear neutrophil elastase were significantly increased in diabetic patients with retinopathy. These markers are significant independent risk factors for retinopathy (Park et al., 2016). A subsequent study injected IL-8 or TNF-α into mouse eyes and found neutrophil infiltration that produced NETs. This is evidenced by immunohistochemically positive staining of NE, MPO, and citH3. These markers were also elevated in vitreous samples from patients with PDR and significantly correlated with their severity (Barliya et al., 2017). NE has been shown to contribute to vascular injury in DR (Liu et al., 2019). There is a large body of data linking NETs to inflammatory kidney disease (Odobasic et al., 2007; Lood et al., 2016; Liu and Dong, 2018). A recent study found increased NET deposition in glomeruli in diabetic nephropathy patients and diabetic mice. The predisposition of diabetic mice to glomerulopathy and glomerular endothelial cell (GEC) damage was attenuated by degrading NETs with DNase I (Zheng et al., 2022). Diabetes predisposes neutrophils to the formation of NETs, which impairs wound healing (Wong et al., 2015). Proteomic analysis of diabetic plantar ulcers in the unhealed group showed enrichment of NET-related proteins, including NE, histone H4, and neutrophil proteases. In addition, this study found a positive correlation between protease-3 and the odds of wound infection (Fadini et al., 2016a). CitH3 was identified as a risk factor for impaired wound healing and amputation (Yang et al., 2020).

Although a formal role for neutrophils in the pathogenesis of T2DM has been demonstrated in preclinical modules of diabetes, further studies in humans are required to elucidate whether neutrophils drive the development of T2DM or whether they are a consequence of obesity and hyperglycemia. The controversy over the generation of NETosis in the pathogenesis of T2DM may be due to the heterogeneity of NETs. NETosis may be associated with patient subgroups representing specific T2DM endotypes. Different types of NETs arise in response to different stimuli, have different components, may contribute differently to diabetic complications, and respond differently to anti-NET therapies. Therefore, researchers still need to continue to investigate the exact mechanisms by which NET components contribute to T2DM and its complications.

### 3.3 Nonalcoholic steatohepatitis

Nonalcoholic steatohepatitis (NASH) is an inflammatory subtype of nonalcoholic fatty liver disease (NAFLD), and it is associated with steatosis as well as hepatocellular injury and inflammation accompanied by varying degrees of liver fibrosis. Accumulation of fat in hepatocytes occurs when fat input or synthesis exceeds fat output or degradation, which is known as steatosis (Machado and Cortez-Pinto, 2014). Triglycerides are the most obvious type of fat in fatty liver (Puri et al., 2007); however, they are not inherently hepatotoxic (Yamaguchi et al., 2007). Other types of lipids that accumulate in the fatty liver may damage hepatocytes (Puri et al., 2007). Hepatocyte damage and death are key features of NASH (Younossi et al., 2011). Damaged hepatocytes can release factors that promote the accumulation of immune cells, produce hepatotoxic substances, and trigger further injury and inflammation. Conversely, factors that promote inflammation also increase hepatocyte exposure to cytokines, gut-derived products, and other inflammatory mediators with hepatotoxic properties (Arrese et al., 2016; Heymann and Tacke, 2016).

Several studies in humans and mice point to a role for neutrophils in NASH. Hepatic neutrophil infiltration is an early event in NASH and can promote inflammation and liver injury (Zhao et al., 2020). Circulating neutrophils correlate with the severity of NASH patients (Antonucci et al., 2020). Patients with NAFLD showed elevated serum NE concentrations and reduced levels of its inhibitor α1-antitrypsin A1AT, resulting in an NE/A1AT imbalance, a ratio associated with NASH progression (Zang et al., 2016). In an NE knockout mouse model fed a Western diet, hepatic steatosis and inflammation are reduced, and NASH is ameliorated (Chen et al., 2019). In addition to NE, plasma MPO levels were higher in NASH mice. MPO deficiency attenuated NASH progression and reduced HFD-induced liver inflammation and fibrosis. This result also suggests an important role of neutrophils in NASH (Rensen et al., 2012).

Several studies have shown that levels of MPO-DNA, markers of NETs, are elevated in patients with NASH and in mouse models of NASH (van der Windt et al., 2018; Du et al., 2022). Another study also found significantly elevated levels of NET markers in the plasma of NASH patients. In addition, this study demonstrated that NETs enhance procoagulant activity in NASH patients (Du et al., 2022). NETs are formed, and the process of NETosis is prolonged in mice fed an MCD-HFD diet for 3 weeks. NETs have also been detected in clinical NASH patients. Inhibition of NETosis can alleviate liver inflammation and liver injury (Wu et al., 2023). In addition, this study demonstrated that NETs did not directly kill neighboring hepatocytes; however, they triggered inflammatory responses in macrophages by upregulating the expression of inflammatory cytokines, including IL-1b, IL-6, and TNF (Wu et al., 2023). A study showed that the progression of hepatocellular carcinoma was also reduced after NETosis inhibition in mice (van der Windt et al., 2018).

### 3.4 Atherosclerosis

NETs are involved in the formation of arterial thrombosis and venous thrombosis (Baptista de Barros Ribeiro Dourado et al., 2022). Platelets are the most important cells involved in hemostasis and thrombosis. A growing body of literature now expresses the view that there is a complex interaction between neutrophils, platelets and NETs in relation to coagulation (de Bont et al., 2019). NETs act as an adhesion meshwork where platelets are recruited and activated by histones on the net, attaching to the net and to the fibrin coagulation meshwork with larger pore sizes, which becomes the basis for thrombus formation. In turn, fibrin clots mixed with NETs are more resistant to fibrinolytic enzymes in the fibrinolytic system, and NETs are themselves prevented from degrading NET structures due to associated complement binding, such as C1q, by the endogenous DNA lysozyme DNase I, exacerbating the formation of thrombi and associated disease (Joiner et al., 1984; Fujihara et al., 2012; Berends et al., 2014; Morgan, 2016; Dhawan et al., 2021).

In contrast, atherosclerosis model mice show that damage to vascular smooth muscle cells induces neutrophil migration, leading to NETosis production (Silvestre-Roig et al., 2019). In contrast, histone H4 in NETs damages smooth muscle cells, leading to plaque instability and an increased risk of detachment (Silvestre-Roig et al., 2019). NETosis is also exacerbated by atherosclerosis, and the main key points of action may be related to oxidized low-density lipoprotein (ox-LDL) and cholesterol crystals (Awasthi et al., 2016). ox-LDL is produced by ROS and has a pro-NET effect. In turn, NETosis, which is stimulated by cholesterol crystals, prompts macrophages to synthesize precursor molecules for IL (interleukin)-1β (Awasthi et al., 2016). At the same time, cholesterol crystals are endocytosed by macrophages, thereby promoting the maturation of endogenous IL-1β, which is thought to be an intracellular mediator of ox-LDL-induced NETosis. Meanwhile, multiple mechanisms of plaque formation have been shown in case studies related to atherosclerosis to have possible involvement of NETs (Warnatsch et al., 2015). NETs contain related components, such as carbon monoxide synthase, NADPH oxidase and MPO, that oxidatively modify high-density lipoprotein (HDL), affecting the body’s lipid-regulating function and contributing to plaque formation (Warnatsch et al., 2015).

## 4 Potential targeted drugs

Targeting NETosis therapy is currently the focus of research. Most targeted drugs are still only attempting to target COVID-19 (Zhu et al., 2022). However, there are also a few experiments trying to explain the possibility of modulating NETosis in obesity and its related diseases. This review subsequently explores the targeted therapeutic strategies that are currently being attempted for the treatment of obesity and its related diseases (Table 1).

**TABLE 1:** Drugs for the treatment of obesity and its related metabolic diseases by targeting NETosis.

Type	Drugs	Model	Pathway	Results	Ref
Obesity	Cl-amidine	diet-induced obesity (DIO) mouse	cathelicidin-related antimicrobial peptide↓	NETosis↓; restored endothelium-dependent vasodilation to the mesenteric arteries	Wang et al. (2018a)
	4-octyl itaconate (4-OI)	normal (lean) or obese mice	Hif-1α↓; HO-1↑	NETosis↓	Burczyk et al. (2022)
T2DM	H2S	LepRdb/db mice and an *in vitro* NETosis model induced byphorbol 12-myristate 13-acetate (PMA) in isolated neutrophils	ROS↓; MAPK↓; ERK1/2 and p38↓	primes diabetic wound to heal	Yang et al. (2019)
	Clarithromycin	Peripheral blood neutrophils obtained from treatment-naive hyperglycemic T2D patients (naive), normoglycemic T2D patients under antidiabetic treatment (well-controlled) and healthy donors (controls)	LL-37↑	antibacterial defense↑; wound healing capacity of fibroblasts↑	Arampatzioglou et al. (2018)
	Vitamin D3/omega-3 PUFAs	Patients and healthy subjects with vitamin D3 deficiency	Neutrophils↑	wound healing in T2DM patients↑; the incidence and severity of complications↓	Basyreva et al. (2021)
	Diethylcarbamazine (DEC)	Polymorphonuclear cells from both healthy and T2D human volunteers	—	NETosis↓	Segoviano-Ramirez et al. (2020)
	Disulfiram	*In vitro* experiments and *in vivo* mouse models of diabetic wound healing	NLRP3/Caspase-1/GSDMD pathway↓	NETs-mediated diabetic foot ulcer healing↓	Yang et al. (2023)
Nonalcoholic Steatohepatitis	Silybin	NASH mice	LA/GLA↓	NETosis↓	Wu et al. (2023)
	Tanshinone IIA (TIIA)	NASH mice	MPO and CitH3↓; caspase-3 and Bax↑	NETosis↓	Xu et al. (2022)
Others	PAD4 inhibitor GSK484	Mice	PAD4↓	endothelial continuity↑	Molinaro et al. (2021)
	Metformin	C57BL/6 mice	High Mobility Group Box-1 (HMGB1)↓	obesity-driven aggressiveness of cancer↓	Chen et al. (2022b)
	Metformin	C57BL/6 mice	High Mobility Group Box-1 (HMGB1)↓	obesity-driven aggressiveness of cancer↓	Chen et al. (2022a)

Cl-Amidine, an inhibitor of peptidyl arginine deiminase 4 (PAD4), specifically inhibits Net (Willis et al., 2011; Knight et al., 2013), and its action is required for histone citrullination during NET formation (Causey et al., 2011; Leshner et al., 2012; Rohrbach et al., 2012). In studying the effects of obesity-associated endothelial dysfunction, researchers evaluated endothelium-dependent vasorelaxation function with angiotensin-converting enzyme (Ach) using a diet-induced obesity (DIO) mouse model. The concentration of plasma monocyte chemotactic protein-1 (MCP-1) was elevated after DIO induction, and Ach-induced vasorelaxation in small mesenteric arteries was impaired. Mice in the DIO group had significantly higher levels of cathelicidin-related antimicrobial peptide (CRIMP), a marker of NET formation, than mice in the standard chow group. Obese mice were treated with Cl-amidine for 2 weeks, and ACh-induced vasorelaxation was significantly improved in DIO mice on chloropyrimidine compared with untreated DIO mice. Therefore, targeting NET formation by Cl-squint may be a feasible approach to treating obesity-associated endothelial dysfunction and inflammation (Wang et al., 2018a).

Itaconic acid is produced endogenously by macrophages in the “broken Krebs cycle” as an intermediate metabolite of lipopolysaccharide processing (Strelko et al., 2011). By exogenously adding 4-octyl itaconate (4-OI), a derivative of itaconate, to normal (lean) or obese mice, researchers found that hypoxia-inducible factor-1α (Hif-1α) was inhibited and heme oxygenase (HO-1) was induced, reducing the formation of Net in normal (lean) or obese mice. This confirms the importance and potential of itaconic acid as a target for NET formation. Detailed testing of the metabolites *in vivo* animal disease models is still needed (Burczyk et al., 2022).

Hydrogen sulfide (H_2_S) is widely recognized as an endogenous signaling molecule, and many of its actions are related to antioxidants (Zhao et al., 2013). H_2_S inhibits the mitogen-activated protein kinase (MAPK) pathway activated by oxidative and reactive oxygen species (ROS) in H9c2 cardiomyogenic myocytes (Dong et al., 2012). Furthermore, in aspirin-treated gastric tissues, H_2_S inhibited neutrophil aggregation and activation, as well as MPO and ROS production (Yang et al., 2017). In a recent study, researchers used an analog of PKC activation by endogenous diacylglycerol (DAG) to treat human neutrophils with phorbol 12-myristate 13-acetate (PMA), which is significantly increased in diabetic conditions, as a model and found that H_2_S pretreatment in the form of Na_2_S resulted in elevated ROS levels. In addition, H_2_S treatment blocked PMA-induced ERK1/2 and p38 phosphorylation, confirming that H_2_S treatment could inhibit NET metabolism and NET release from diabetic wounds, thus promoting wound healing and providing clues for H_2_S targeting NETosis for the treatment of diabetic skin complications. However, in this study, the *in vitro* study model could not fully mimic the real NETosis process in diabetic mice or patients. This may be a limitation given the differences between humans and mice (Yang et al., 2019).

The macrolide antibiotic clarithromycin has been shown to be a potential immunomodulator and a known inducer of LL-37-bearing NETs (Konstantinidis et al., 2016). A recent study further demonstrated its ability to induce antimicrobial activity in diabetic NET structures *in vitro*. Researchers collected peripheral blood neutrophils from hyperglycemic T2DM patients at the beginning of treatment (NAIVE), normoglycemic T2DM patients after glucose-lowering therapy (well-controlled), and healthy blood donors (controls) and performed *E. coli* NCTC 9001 cultures in the presence or absence of neutrophils from naive and well-controlled T2DM patients. After stimulation of NET production with clarithromycin *in vitro*, LL-37 was found to be significantly more externalized on NET produced by well-controlled T2DM neutrophils. This suggests that clarithromycin may enhance the antimicrobial defense and wound-healing capacity of fibroblasts by upregulating LL-37 expression on NET structures, which may provide a further advantage to patients with well-controlled T2D (Arampatzioglou et al., 2018).

Data reported in the literature suggest that vitamin D3/omega-3 PUFA supplementation significantly reduces ROS/RHS production and increases total serum antioxidant capacity (Razzaghi et al., 2017; Soleimani et al., 2017; Lu et al., 2018; Heshmati et al., 2019). A recent study was conducted by combining vitamin D3 with omega-3 PUFAs (vitamin D3/omega-3 PUFAs) in T2DM patients with septic necrotizing lesions of the lower extremities and healthy subjects, both of whom are deficient in vitamin D3. Using phorbol-12-myristate-13-acetate (PMA) activation of neutrophils in isolated blood as a model of NETosis, it was found that after *in vitro* whole blood PMA activation of neutrophils, administration of vitamin D3/omega-3 PUFAs completely “turned off “NETosis and neutrophil death. This suggests that supplementation with vitamin D3/omega-3 PUFAs reduces the ability of neutrophils to produce NETs and has a positive effect on wound healing in patients with T2DM, reducing the incidence and severity of complications. However, this preliminary study included only a small number of patients and healthy subjects and should be followed by a double-blind placebo-controlled study with a sufficiently large number of participants (Basyreva et al., 2021).

Decamazin (DEC), a water-soluble derivative of piperazine, is an antiparasitic drug. Some studies have reported the dose-dependent immunomodulatory effects of DEC on phagocytosis in animal models (García-Hernández et al., 2014; Medina-De la Garza et al., 2012). In a recent study, researchers incubated purified polymorphonuclear cells (PMNs) from both healthy and DM2 volunteers with DEC followed by induction of NETosis with PMA and administered DEC 1 h prior to the PMA challenge. It was found that DEC had an immunomodulatory effect in both healthy and DM2 populations by reducing and delaying the formation of NETs from PMNs. This phenomenon reveals that DEC may have clinical application in the control of wounds and plantar ulcers in DM2 patients and provide them with benefits in terms of treatment cost and quality of life (Segoviano-Ramirez et al., 2020).

Disulfiram has been proven to inhibit acetaldehyde dehydrogenase and has been used to treat alcoholism (Koppaka et al., 2012). Previous studies have shown that neutrophils use inflammatory vesicles and GSDMD-dependent mechanisms to activate NETosis as a defense response against cytoplasmic bacteria (Chen et al., 2018). Disulfiram is a potent inhibitor of GSDMD (Hu et al., 2020). In a recent study, researchers found that inhibition of GSDMD by disulfiram eliminated NET formation and thus accelerated diabetic wound healing through *in vitro* experiments and *in vivo* diabetic wound healing mouse models. The possible mechanism is through inhibition of the NLRP3/Caspase-1/GSDMD pathway, thereby inhibiting NETs-mediated diabetic foot ulcer healing injury (Yang et al., 2023).

Silybin, derived from silymarin, has multiple pharmacological effects and may alleviate nonalcoholic steatohepatitis (NASH) by reducing lipid accumulation (Sun et al., 2020; Zhang et al., 2021b). In a recent study, researchers intragastrically gavaged (ig) with vehicle or 50 mg/kg silybin every day since the induction of NASH. The results revealed for the first time that silybin-pretreated neutrophils were resistant to linoleic acid (LA)- or γ-linolenic acid (GLA)-mediated NETosis, possibly due to the inhibition of ROS production. Silymarin also inhibited the formation of NETs in NASH animals and alleviated MCD-HFD-induced liver inflammation, steatosis and fibrosis. These results suggest that silybin can alleviate inflammation in NASH while inhibiting NETs. This demonstrates its potential as an important inhibitor of NETs and its therapeutic potential in the treatment of NETosis-related diseases (Wu et al., 2023).

Tanshinone IIA (TIIA) is one of the main active components of the traditional Chinese medicine Salvia miltiorrhiza Bunge. It has been reported to have several pharmacological effects (Tan et al., 2019; Fang et al., 2020b; Fu et al., 2021) and has been widely used in the treatment of cardiovascular diseases (Ansari et al., 2021). Recently, some researchers investigated the therapeutic effects and mechanisms of TIIA on methionine choline deficiency (MCD) diet-induced nonalcoholic steatohepatitis (NASH) in mice. TIIA attenuated inflammatory progression and reduced hepatocyte apoptosis in a mouse model by inhibiting the formation of MPO and CitH3 in the neutrophil extracellular trap network and suppressing caspase-3 and Bax-mediated apoptosis in hepatocytes. These results suggest the potential applicability of TIIA as a therapeutic agent for NASH (Xu et al., 2022).

In the treatment of atherosclerosis, some researchers have developed collagen type IV-targeted nanoparticles (Col IV NPs) that selectively deliver PAD4 inhibitors to areas of endothelial cell collapse and type IV collagen-rich basement membrane exposure. Delivery of the PAD4 inhibitor GSK484 reduced NET accumulation at sites of endothelial injury and maintained endothelial continuity. This result demonstrates a new therapeutic approach to limit plaque erosion in endothelial injury and further supports the role of PAD4 and NETs in superficial erosion, suggesting that the PAD4 inhibitor GSK484 may be a targeted agent for the treatment of atherosclerosis (Molinaro et al., 2021).

In addition to the known hypoglycemic and antidiabetic effects of metformin, studies have shown that metformin has other therapeutic applications in terms of anticancer potential (Zhang et al., 2021a; Kang et al., 2021; Sun et al., 2022). In a recent study, researchers investigated the anticancer effects of metformin using a homologous tumor model established in mouse LLC cells and C57BL/6 mice fed a high-fat diet with obese mice. The results revealed that data from neutrophil studies confirmed the inhibitory effect of metformin on HMGB1-induced network formation. In addition, HMGB1 was identified as a facilitator molecule that promotes the transition process to NETS. These results suggest that metformin plays a role in obesity-induced cancer aggressiveness by inhibiting HMGB1-induced NET formation and may be a potential target drug (Chen et al., 2022b).

## 5 The role of lifestyle, diet, microbiome, genetics, and environmental factors

NETosis is mediated not only by drugs but also by a variety of other factors. Lifestyle, diet, microbiome, genetics, and environmental factors have been found to play multiple roles in regulating NETosis in obesity and its related diseases and are therefore explored in the following sections. factors are discussed below.

In recent years, the impact of lifestyle-derived risk factors on NETosis has received increasing attention. Poor lifestyles such as unbalanced diet, physical inactivity, smoking, and inadequate sleep quality are associated with NETosis and increase the risk of related diseases. Lack of regular physical activity affects whole immune system function. It has been shown that the levels of lactic acid (LA) can increase up to 20-fold in blood plasma after an acute bout of exhausting exercise (Abebayehu et al., 2016). LA negatively regulates neutrophil NET capacity. *In vitro*, increased LA concentrations are also associated with reduced rates of NET release and decreased ROS formation (Shi et al., 2019). It is well known that smoking induces oxidative stress in endothelial cells. However, it was recently proposed that smoking also affects the function of immune cell populations. Studies have shown that exposure of mouse and human neutrophils to chronic tobacco smoke induces massive NET formation (Qiu et al., 2017; Albrengues et al., 2018), and neutrophils isolated from smoking subjects showed elevated rates of spontaneous and PMA-induced NETosis (Lee et al., 2017a). Isolated human neutrophils are capable of NETosis via nicotine-stimulated nicotinic acetylcholine receptor (nAChR) stimulation, which may exacerbate inflammatory pathways and induce tissue damage (Hosseinzadeh et al., 2016). Sleep deprivation is believed to be an important risk factor for immune disorders. It has been proven that shortened sleep duration and sleep fragmentation in humans increase neutrophil and monocyte counts in blood and peripheral tissues (Beiter et al., 2014; Carreras et al., 2014; Vallat et al., 2020). However, there are no scientific reports delving into the link between NETs and sleep deprivation, and the mechanisms linking the two remain to be investigated.

High-fat diets have been noted to be associated with NET formation. Studies have shown that neutrophils from high-fat diet (HFD)-fed mice are more likely to spontaneously form NETs in the lungs than neutrophils from low-fat diet-fed animals (Moorthy et al., 2016). Feeding an HFD induces neutrophil recruitment into adipose tissue and increases NET formation (Moorthy et al., 2016; Wang et al., 2018a). NOD1 in HFD-fed mice drives NETosis, which contributes to the regulation of atherogenesis progression (Fernández-García et al., 2022). In recent years, the gut microbiota was found to be strongly associated with NETosis formation. Gut microbiota and its metabolite butyrate significantly reduced neutrophil infiltration and NOX2-dependent NETosis, inflammation, and abnormal phenotypic switching of vascular smooth muscle cells in the aortic wall, which resulted in significant attenuation of abdominal aortic aneurysm development (Tian et al., 2022). In addition, oral butyrate significantly improves and ameliorates mucosal inflammation in inflammatory bowel disease by inhibiting NET formation (Li et al., 2021). It has also been found that gut flora can exacerbate cardiac ischemia/reperfusion (I/R) injury by modulating the formation of NETs (Chen et al., 2022a). Gut flora inhibits NET neutrophil hyperreactivity in mesenteric I/R injury (Ascher et al., 2020).

Increasing evidence suggests that single nucleotide polymorphisms (SNPs) play an important role in the formation of NETs. It was found that neutrophil ROS and NET formation are regulated by the NCF1-339 genotype and that the NCF1-339 SNP-mediated decrease in NADPH oxidase function is associated with high interferon activity and impaired formation of NETs in SLE, permitting a dependence on mitochondrial ROS (Linge et al., 2020). In another study, the C1858T SNP was found to be associated with an increased tendency to form NETs in peripheral blood monocytes with excess citrulline and an increased propensity for spontaneous NET formation and increased the risk of RA by enhancing the spontaneous formation of protein citrulline and NETs (Chang et al., 2015). LNK (R262 W) decreases LNK function in human platelets and neutrophils, promotes NETosis, and increases the risk of coronary artery disease in carriers (Dou et al., 2021). Mendelian randomization is also likely to explain the causal relationship between NETosis and obesity.

The role of environmental factors in regulating NETosis should not be overlooked. Chronic exposure to high levels of fluoride can lead to severe neuropathological damage in the brain. A recent study has shown that fluoride leads to the opening of calcium channels by causing a calcium imbalance in neutrophils, which in turn leads to the opening of L-type calcium channels (LTCCs). Extracellular free iron enters the cell from the open LTCC, leading to neutrophil iron death, which releases NETs and exacerbates neuronal cell inflammation (Wang et al., 2023). UVA and UVB radiation have been shown to induce NET formation in human polymorphonuclear cells (Zawrotniak et al., 2019). NET formation is activated not only by UV-A but also by the three visible light spectra, blue, green, and orange, in a dose-dependent manner. Light-induced NETosis was found to proceed via NADPH oxidase and PAD4 (Arzumanyan et al., 2023). Furthermore, it has been revealed that in an asthma model, PM2.5 aggravates NQO1-induced mucus hypersecretion through the release of NETs (He et al., 2021).

In conclusion, the existing research on the regulation of NETosis in obesity and its related diseases has shed light on the multifaceted roles played by lifestyle, diet, microbiome, genetics, and environmental factors. However, it is important to note that there are certain limitations and gaps in our current understanding. Most studies have primarily focused on individual factors and their impact on NETosis regulation. Future research should aim to investigate the complex interplay and synergistic effects of these factors, as they are likely to interact and influence NETosis in a more comprehensive manner. In the future, a multidisciplinary approach integrating genetics, microbiome, immunology, and environmental sciences will be essential to unravel the complexities of NETosis regulation. This will enable the development of personalized interventions and therapeutic strategies for individuals suffering from obesity and its associated diseases.

## 6 Conclusion

Extensive research has been conducted on the discovery of NETosis, its mechanism, and its effects on various diseases. Presently, three principal mechanisms of NETosis have been recognized: NOX-dependent NETosis, non-NOX-dependent NETosis, and rapid production-release NETosis. The formation of different types of NETosis may have varying effects depending on the scenario. Although NETosis can be beneficial by entrapping and eliminating pathogens, there is increasing evidence indicating that it also plays a crucial role in diseases such as obesity, T2DM, and NASH. Excessive activation and inappropriate recruitment of NETosis may cause severe organismal damage and disease progression.

However, current research on NETosis is limited, and many challenges remain that require attention. For example, in the study of NEtoSis-related diseases, a standardized method for detecting NETosis is needed. In addition, many clinical studies have small samples, and it is necessary to collect more clinical evidence to confirm the universality of the pathogenic effects of NETosis. In addition, the mechanism of the interaction between NETosis and chronic metabolic diseases is not fully understood, and there is a lack of complete mechanistic studies. Future studies should focus on clarifying and improving the investigation of the mechanism of NETosis in various diseases and how to control the generation and interruption of NETosis. These mechanisms.

As research on NETosis continues to advance, controlling NETs is rapidly becoming a therapeutic target for managing a wide range of diseases. Therefore, the importance of developing targeted drugs cannot be overemphasized. These drugs can regulate NETs by disrupting the formation of NETs or by targeting compounds in NET components such as NNA or NE. Therefore, when developing NETosis-targeted drugs, attention must be paid to the types of NETosis stimulation and changes in signaling pathways under different pathological conditions to more specifically target the disease. It is important to note that the phagocytosis of neutrophils is central to host defense, and any targeted drug against NET formation must avoid impairing the physiological function of these cells. Clinical trials could be designed to test the efficacy of NET interfering drugs in obese patients.
